# A high-throughput screen to identify novel synthetic lethal compounds for the treatment of E-cadherin-deficient cells

**DOI:** 10.1038/s41598-019-48929-0

**Published:** 2019-08-29

**Authors:** Henry Beetham, Augustine Chen, Bryony J. Telford, Andrew Single, Kate E. Jarman, Kurt Lackovic, Andreas Luxenburger, Parry Guilford

**Affiliations:** 1 Cancer Genetics Laboratory, Department of Biochemistry, University of Otago, Dunedin, New Zealand; 2 Division of Systems Biology and Personalized Medicine, Walter and Eliza Hall Institute of Medical Research, Parkville, Victoria Australia; 3 Department of Medical Biology, University of Melbourne, Victoria, Australia; 4 Ferrier Research Institute, Victoria University of Wellington, Lower Hutt, New Zealand

**Keywords:** Breast cancer, Gastrointestinal cancer, Cadherins, High-throughput screening, Phenotypic screening

## Abstract

The cell-cell adhesion protein E-cadherin (*CDH1)* is a tumor suppressor that is required to maintain cell adhesion, cell polarity and cell survival signalling. Somatic mutations in *CDH1* are common in diffuse gastric cancer (DGC) and lobular breast cancer (LBC). In addition, germline mutations in *CDH1* predispose to the autosomal dominant cancer syndrome Hereditary Diffuse Gastric Cancer (HDGC). One approach to target cells with mutations in specific tumor suppressor genes is synthetic lethality. To identify novel synthetic lethal compounds for the treatment of cancers associated with E-cadherin loss, we have undertaken a high-throughput screening campaign of ~114,000 lead-like compounds on an isogenic pair of human mammary epithelial cell lines – with and without *CDH1* expression. This unbiased approach identified 12 novel compounds that preferentially harmed E-cadherin-deficient cells. Validation of these compounds using both real-time and end-point viability assays identified two novel compounds with significant synthetic lethal activity, thereby demonstrating that E-cadherin loss creates druggable vulnerabilities within tumor cells. In summary, we have identified novel synthetic lethal compounds that may provide a new strategy for the prevention and treatment of both sporadic and hereditary LBC and DGC.

## Introduction

E-cadherin is a calcium-dependent transmembrane glycoprotein, expressed predominantly at the adherens junction on the basolateral surface of epithelial cells. It has long been regarded as a tumor suppressor due to its frequent downregulation in sporadic tumors during invasion and metastasis^1^ and the high frequency of inactivating *CDH1* mutations that are observed in diffuse gastric cancer (DGC) and lobular breast cancer (LBC). E-cadherin loss, caused by mutations or epigenetic silencing, contributes to a loss of cell polarity, increased migration and the epithelial-mesenchymal transition (EMT)^2,3^.

Hereditary Diffuse Gastric Cancer (HDGC) is characterized by multiple foci of stage T1a signet ring cell carcinoma that develop in the stomachs of *CDH1* mutation carriers following the downregulation of the 2nd *CDH1* allele^4,5^. A few percent of all gastric cancers are defined as HDGC and 30–50% of patients meeting the clinical criteria for HDGC have germline *CDH1* mutations^6,7^. Individuals from HDGC families have a ~70% lifetime risk of developing DGC^8,9^. Females with germline *CDH1* mutations have an additional ~40% lifetime risk of developing LBC^8,10,11^.

Breast cancer is both the most common cancer in women and the leading cause of cancer death^12^. The loss of E-cadherin expression is more common in the lobular- rather than ductal-type carcinoma of the breast^13,14^ and has been observed in up to 90% of cases. Between 10–15% of breast cancers diagnosed are LBC^15^. Although E-cadherin loss is found in a range of sporadic cancers^10,16–18^, LBC is the only non-gastric cancer over-represented in families with HDGC^19,20^.

Prophylactic total gastrectomy is currently the safest treatment option for germline *CDH1* mutation carriers^9,21^, although nearly all post-gastrectomy patients develop complications resulting from surgery and about one third of patients have major adverse events^22–27^. The breast cancer risk in HDGC families is usually managed by routine screening; prophylactic mastectomy is currently not recommended^9^, but remains an option for some women. Prophylactic mastectomies are however, common in women with lobular carcinoma *in situ* (LCIS)^28^. LCIS is a LBC precursor that is often *CDH1* negative and increases the risk of developing LBC by 8–10 fold^28,29^.

As these cancers are characterised by the absence of E-cadherin, conventional drug targeting cannot be used. We propose that the loss of E-cadherin in early stage DGC and LBC could be specifically targeted using a synthetic lethal (SL) approach. Synthetic lethality is classically defined as a genetic interaction in which a combination of mutations in two or more genes - but not each gene alone - leads to cell death. In a therapeutic setting the term can refer to the use of a targeted drug to cause cell death exclusively in tumors carrying specific genetic alterations.

By eliminating E-cadherin-negative precancerous cells before they have an opportunity to progress, novel compounds may provide an alternative approach to the prevention of DGC and LBC in HDGC families. In addition, these E-cadherin SL drugs could provide a new option for the treatment of LCIS and advanced DGC and LBC.

In order to study chemoprevention in an early disease model of HDGC, MCF10A was selected as a ‘normal’ non-malignant adherent epithelial cell line^30,31^. We have previously characterized the E-cadherin-null isogenic partner of MCF10A (MCF10A *CDH1*^*−/−*^) and shown it to affect cell morphology, migration capabilities and cell adherence, but anchorage-dependent growth and cell-cell contacts were maintained. The MCF10A *CDH1*^*−/−*^ cells show an increased ‘cancer-like’ phenotype, but still remain relatively indolent^3^. Importantly, E-cadherin loss is the first step in the development of HDGC, and therefore an appropriate chemoprevention target despite the absence of cell transformation.

To identify novel SL compounds for the treatment of cancer arising from E-cadherin loss, we screened the Stage 6 WECC library of 113,945 novel lead-like compounds^32^. These compounds were selected from a possible 4.9 million novel compounds, based on gold standard lead-like criteria^32,33^. Lead-like compounds are simpler, more polar and smaller than drug-like compounds (Supplementary Fig. 1). In addition, no compound was more than 85% similar to any other as judged by the Tanimoto coefficient. This ensured that the library did not have large numbers of highly similar analogues and that classical reactive and non-drug-like compounds were removed. These criteria allow for attractive and optimisable starting points for further development.

Four distinct screening and validation stages were undertaken using the WECC library (Supplementary Fig. 1); (i) an assay miniaturization and pilot screen, (ii) primary screen, (iii) single-point confirmation, and (iv) an 11-point dose-response screen for EC_50_ determination.

## Results

### Pilot screen

A pilot screen of 10,208 WECC compounds randomly selected from the 113,945 WECC novel compound library was screened in two biological replicates using the MCF10A *CDH1*^*−/−*^ cell line. This initial stage was used to characterise and reduce edge effects, assess if the compound concentration of 10 µM resulted in an acceptable hit rate (<3%) and determine the plate-to-plate reproducibility. As we have previously shown a SL interaction with entinostat^34^, an EC_50_ dose was used as a SL control and an EC_80_ dose of doxorubicin was used as a killing control (Supplementary Table 1).

#### Practical mitigation of positional effects

**Figure 1:**
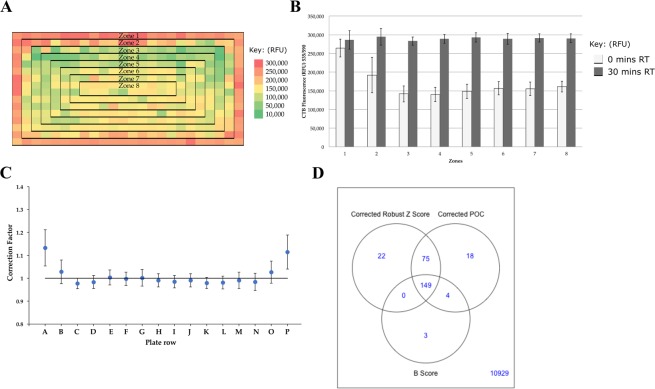
Mitigating positional effects. (**A**) Positional effects due to temperature. An entire 384-well plate was seeded at 800 cells/well. After CTB incubation at 37 °C for three hours, the plate was read immediately in the EnVision (0 minutes RT). This edge effect is shown with white bars in B. (**B**) Thermal gradients between plate zones have a significant effect on fluorescence readings for MCF10A cells. After CTB incubation the plate was left for 0 minutes RT before reading (white bars) and then left for 30 minutes RT (black bars) before another reading was taken. Plates were handled as described in the methods section. Data is shown as mean +/− SD. (**C**) The correction factor was used to mitigate systematic edge effects. A data set was created from all pilot and primary screen compound well correction factors. Data is shown as mean +/− SD. (**D**) Comparison of normalization methods to mitigate edge effects. Data is from the pilot screen.

Despite controlling for the usual causes of positional effects in HTS^35^, we found that temperature fluctuations of the plates during end-point cell titre blue (CTB) fluorescence readings were producing a classic edge effect (Fig. 1A). When plates were read immediately after CTB incubation there was a statistically significant decrease in relative fluorescence between zone one (the edge wells) and every other zone of the plate (Fig. 1B). This was mitigated by leaving plates for 30 min to equilibrate to RT. Similar edge effects were observed under conditions of limited evaporation (data not shown).

#### Normalization methods to resolve positional effects

To mitigate systematic edge effects in the pilot and primary screening data, a custom well-correction factor was applied to both the percent of controls (POC; normalizes samples to the DMSO and doxorubicin controls) and the robust Z score (RoZS; uses the samples themselves, as *de facto* controls). This was calculated from the batch median of each well (Supplementary Fig. 3). In practice this led to rows A and P in the pilot screen having a mean well-correction factor of 1.13 and 1.11, respectively (Fig. 1C).

The B score^36^ was also examined. Using the data for the pilot screen and a hit cut-off of 3 × SD below the mean, 55.0% of the hits correlated between all three methods (Fig. 1D). The B score method was more stringent and resulted in only three unique hits, whilst the corrected RoZS and corrected POC had a greater range of unique hits.

The activity of a compound tested at a fixed concentration in one replicate will not reliably predict the true effectiveness of a compound^37^ and hence marginal hits can have good true potency, but may be concealed during the HTS campaign. Therefore, for the combined pilot and primary screens, both the corrected RoZS and corrected POC methodologies were used to identify the widest range of possible hits whilst resolving positional effects.

#### Plate-to-plate reproducibility

Since only one replicate was to be performed at the primary screening stage, the plate-to-plate reproducibility was determined in the pilot screen. The non-parametric Spearman’s rank correlation coefficient was used as a measure of the strength of an association. The mean Spearman correlation between the two biological replicates for the 32 plates assayed in the pilot screen was 0.42, with the lowest being 0.29 and highest 0.57 (data not shown). Of the hits determined by the threshold ‘mean −3 × SD’ for the pilot screen, this medium-strength of correlation resulted in 70% of the replicate one hits overlapping with replicate two hits for corrected RoZS and corrected POC. For replicate two hits, 85% overlapped with replicate one hits for both corrected normalization methods (data not shown). Considering there can be around a 50% rate of ‘false hits’ in primary screens^38^, these overlaps were considered sufficient to progress with the primary screen.

### Exploiting E-cadherin loss

The primary screen was assayed in one biological replicate with an additional 103,737 compounds screened against the MCF10A *CDH1*^*−/−*^ cells. For quality control the Z’ factor was used to ensure that each plate in the pilot and primary screens had a statistically appropriate separation between the positive and negative controls. All 325 assay plates had acceptable Z’ values above 0.4 and 62% of plates had excellent Z’ factors above 0.6.

#### Inclusive hit selection

**Figure 2:**
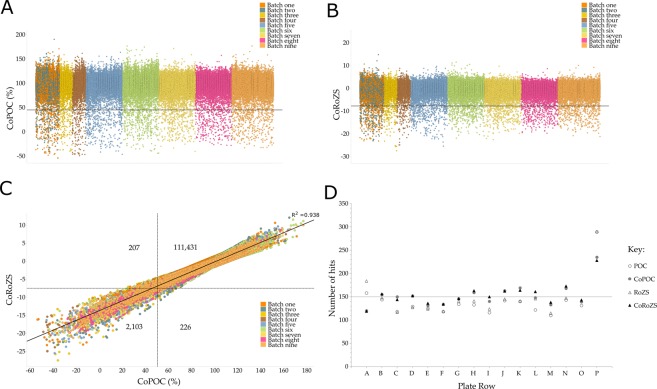
Pilot and Primary Screens. (**A**) Corrected percent of controls (CoPOC). Hits were <44.73% (3 × SD below the mean), leading to 2,310 hits (2.03%). (**B**) Corrected robust Z score (CoRoZS). Hits were <−7.81 (3 × SD below the mean), leading to 2,329 hits (2.04%). (**C**) Hit identification strategy for the pilot and primary screens. Hits from these two normalization methods were combined and the duplicates removed for the hit selection. The number of hits for each normalization method are shown. (**D**) Distribution of hits by row. Hits are from the combined pilot and primary screens using the threshold of mean −3 × SD.

A hit threshold of 3 × SD below the mean for the combined pilot and primary screening data resulted in 2,310 compounds (2.03%) from the corrected POC and 2,329 (2.04%) from the corrected RoZS (Fig. 2A,B). There was a strong correlation (R^2^ = 0.938) between the two normalization methods (Fig. 2C) with an overlap of 82.9%. To avoid removing potentially genuine hits, the hits from these two normalization methods were combined. This resulted in a hit selection of the top 2,536 (2.2%) most active compounds which were below the pre-determined <3% for 10 µM. These top 2.2% of compounds that produced the greatest reduction in viability of MCF10A *CDH1*^*−/−*^ cells were then carried forward to the single point confirmation screen.

#### Positional effects observed in the distribution of hits

Since the WECC library compounds are randomly dispersed throughout the pilot and primary screen plates, each row and column will be expected to have an even distribution of hits (Fig. 2D). The most striking differences were seen in the uncorrected data for edge rows A and P. In row P, there were 149 and 157 more hits than the median hits per row, for RoZS and POC respectively. The corrected data reduced the number of these likely false positive, positional effect hits. For example, row P had 54 less hits for corrected POC compared to POC and 62 less hits for corrected RoZS compared to RoZS (Fig. 2D). Similar trends were seen for the median number of hits per column (data not shown). However, it is likely that even with the correction factor there were false positive hits due to edge effects occurring in row P.

### Identification of synthetic lethal compounds

**Figure 3:**
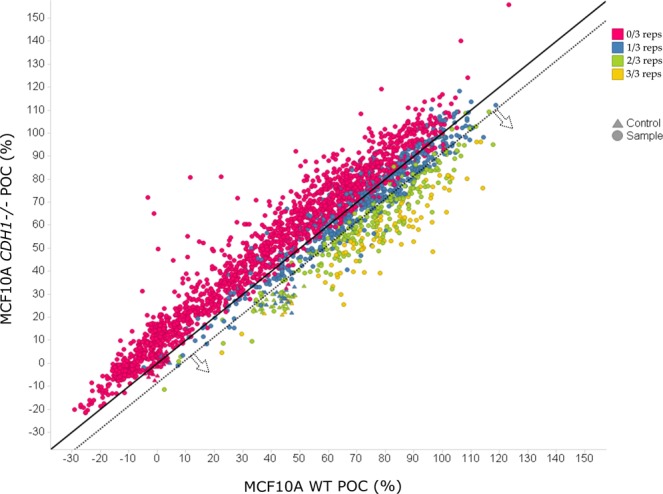
Single-point confirmation screen for MCF10A WT and *CDH1*^*−/−*^ cells. The average differential of each compound is shown as a circle and the doxorubicin and entinostat controls as triangles. The samples in which all three biological replicates have a differential above 8.5% are shown in yellow, those with 2/3 replicates above 8.5% in green, samples with 1/3 replicates above 8.5% in blue, and in red, samples with no replicates with a differential above 8.5%. The bold black line is Y = X, the dotted black line is Y = X-8.5.

In the single-point confirmation screen, compounds that preferentially harm MCF10A *CDH1*^*−/−*^ cells compared to the wild-type cells were now identified. Three biological replicates were performed for each of the isogenic cell lines at a single-point (10 μM) in 384-well plates. All assay plates had acceptable Z’ values above 0.4 and 23 out of 48 plates had values above 0.6. The POC R^2^ correlation between the three biological replicates for the two isogenic cell lines were all above 0.8 (data not shown). A cell viability differential of ‘MCF10A WT POC – MCF10A *CDH1*^*−/−*^ POC’ was used to select hits. An arbitrary threshold for the mean differential of >8.5% was chosen in order to maximize the number of hits being screened in the subsequent stages and to reduce false negatives. This resulted in 308 hits above the mean differential threshold, hence crossing the Y = X-8.5 line (Fig. 3).

To incorporate a measure of the variability between replicates of the screen into the hit selection process, a further requirement was included of a minimum of 2/3 biological replicates having viability differentials >8.5%. This removed a further 52 compounds shown in blue with only one out of three biological replicates crossing the Y = X-8.5 line independent of the mean. In total, 256 compounds were above the hit selection threshold, thereby preferentially killing E-cadherin negative cells. The mean POC differential of the 256 hits was 17.6% +/− 7.1 (+/−SD), with the highest being 48% for SLEC-1 (Synthetic Lethal E-Cadherin compound **1**). The entinostat SL controls had an average POC differential of 15%, and therefore validated the SL hits. The resulting 256 SL compounds were then assayed in an 11-point screen for EC_50_ determination.

Interestingly, there were 1,797 compounds out of 2,536 that had average cell viability differentials that harmed MCF10A WT cells more than the MCF10A *CDH1*^*−/−*^ (Fig. 3). These opposite hits have been termed ‘reverse synthetic lethal’ (RSL) compounds^39^.

**Figure 4:**
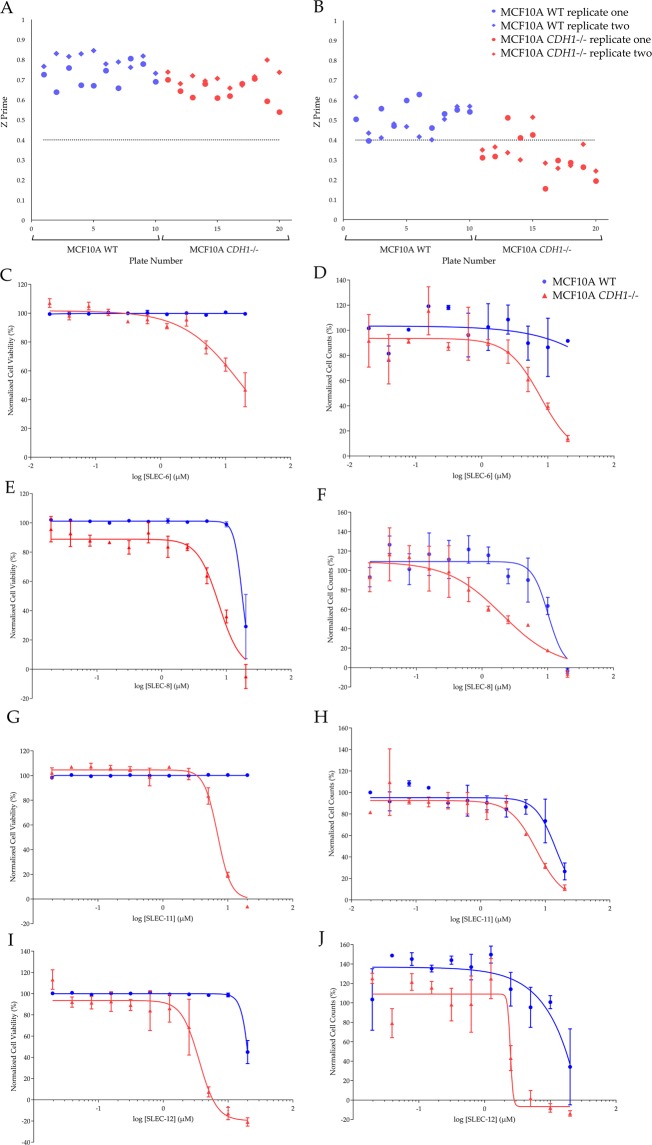
Lead synthetic lethal compounds from the 11-point screens using CTB and HCI for end-point analysis. Normalized cell counts or normalized cell viability were calculated using the POC method. **(A,C,E,G,I**) data is from the CTB screen. (**B,D,F,H,J**) data is from the HCI screen. (**A,B**) Z prime for the two isogenic cell lines. Blue data points represent MCF10A WT, and red represent MCF10A *CDH1*^*−/−*^. (**C–J**) Data shown is the average of two biological replicates, error bars are SEM. Blue lines with circles represent MCF10A WT, and red lines with triangles represent MCF10A *CDH1*^*−/−*^. The base 10 log scale was used. (**C,D**) Dose-response for SLEC-6. (**E–F**) Dose-response for SLEC-8. (**G–H**) Dose-response for SLEC-11. (**I,J**) Dose-response for SLEC-12.

The 11-point dose-response screen evaluated a concentration range from 0.02 µM to 20 µM for both isogenic cell lines in duplicate biological replicates (Fig. 4). One limitation to metabolism-based cellular assays is that a change in read out (fluorescence, luminescence or absorbance) may not necessarily be caused by cell death and could instead result from a cessation of proliferation due to senescence or inhibited mitochondrial respiration. For this reason, a direct measurement of cell counts using cellular imaging of nuclei stained with Hoechst 33342 was also included at the 11-point screening stage. For the CTB 11-point screen all plates had acceptable Z’ values above 0.4, with the second biological replicate having 70% above 0.6 (Fig. 4A). The Z’ values for the high-content imaging (HCI) 11-point screen plates were above 0.4 for MCF10A WT, except one plate from biological replicate two with 0.396 (Fig. 4B). However, for MCF10A *CDH1*^*−/−*^ HCI data, only four plates were above a Z’ of 0.4 and the mean Z’ was 0.32 compared to a mean of 0.51 for MCF10A WT. A further quality metric known as the strictly standardized mean difference (SSMD)^40^ is a less conservative approach for QC and a cut-off of >3 is generally used. The mean SSMD value was 7.04 for MCF10A WT and 5.27 for MCF10A *CDH1*^*−/−*^ plates and indicates the MCF10A *CDH1*^*−/−*^ HCI data was acceptable^41^. There was a good correlation between the top hits from the HCI and CTB 11-point screening with an overlap of 31 / 40 for the top 40 compounds selected from the eventual hit selection strategies.

**Figure 5:**
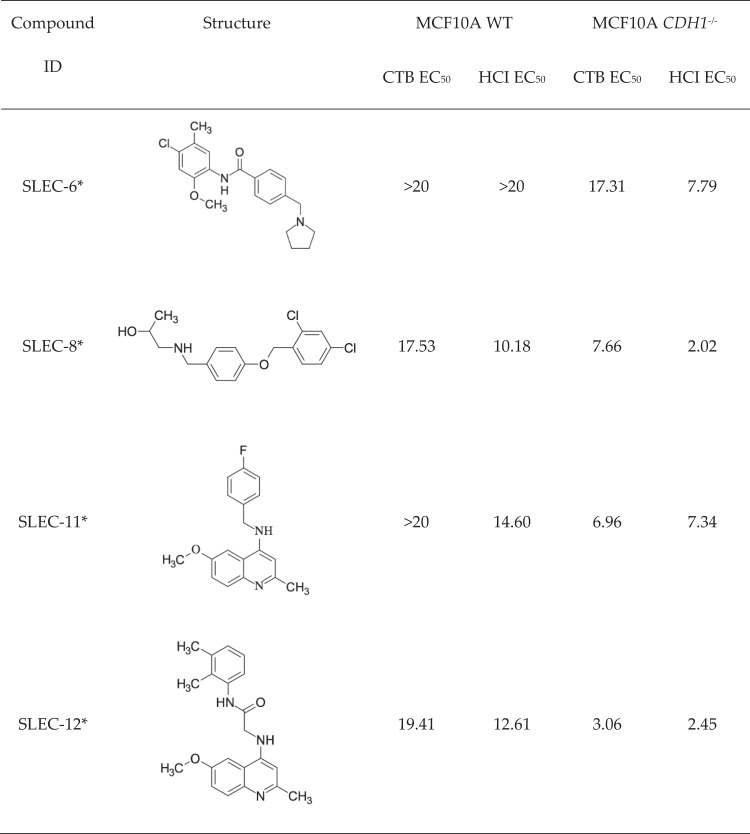
Structures and EC_50_ values for lead compounds. EC_50_ values for the lead compounds for MCF10A WT and MCF10A *CDH1*^*−/−*^ cells calculated from the CTB and HCI 11-point screens.

In general, POC values determined from the HCI screen were lower in both cell lines for the same concentration compared to the CTB screen. SLEC-8 had the lowest HCI EC_50_ value for MCF10A *CDH1*^*−/−*^ cells of 2.02 μM and SLEC-12 had the second lowest CTB EC_50_ value of 3.06 μM (Fig. 5). SLEC compounds 8 and 12 also had the best EC_50_ ratios between MCF10A WT and MCF10A *CDH1*^*−/−*^ cells of 5.03 and 6.34 fold change, respectively.

### The weighted and ranked score

**Figure 6:**
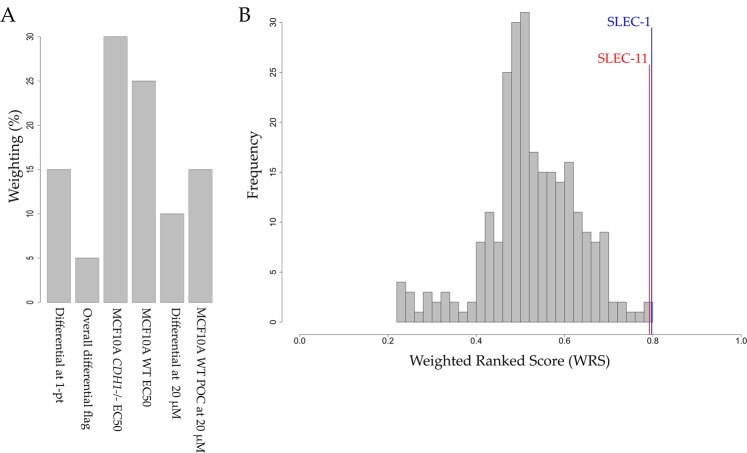
The weighted and ranked score. Only the 256 hits entering the 11-point dose-response screens were considered. Compounds were scored using the formula: variable / max(variable) to give a score from 0–1, with 1 being the ideal score. All data was averaged between the three or two biological replicates, for single-point and 11- screens, respectively. (**A**) Percentage weighting of each variable. (**B**) Distribution of the sum of weighted and ranked variables.

To prioritise hits, a weighted and ranked score (WRS) was created for each compound. Rather than using solely an EC_50_ from each cell line or an extensive list of multi-parametric data from the four-staged screen, six key variables were chosen. Each variable was given a different weighting based on their predicted importance. The sum of the weighted and scored variables produced the WRS to summarise the multi-parametric data of each compound into a single number (Fig. 6). The WRS is a unique tool for hit-selection and was used in combination with a comprehensive assessment of the biological activity of each compound including non-quantifiable traits, i.e. chemical desirability of the scaffold and risks associated with structural features or synthetic accessibility to determine the final lead compounds from the four-staged screen.

For the single-point confirmation screen the two variables used to determine the strength of a SL hit were (i) the POC differential between the two MCF10A cell lines at 10 μM (15% weighting), and (ii) how many of the three biological replicates also had a differential above the 8.5% POC threshold (5% weighting). Since the single point screen was assayed at only one concentration, these two variables were given a combined weighting of 20%.

For the subsequent 11-point screen, the EC_50_ for MCF10A WT and MCF10A *CDH1*^*−/−*^ were given a 25% and 30% weighting, respectively. As well as this, the differential at 20 μM (10% weighting), and the viability of the MCF10A WT cells at the highest concentration of 20 μM (15% weighting) were also included.

The mean WRS was 0.53, with only the top five compounds being >2 × SD from the mean and no compound having a WRS greater than 0.8 (Fig. 6).

Using a subjective triage of the 256 compounds at the 11-point screening stage combined with the WRS as a guide for an objective hit selection, 84 SL hits were selected to identify common pharmacophore groups.

### Determining theoretical pharmacophore groups

The selected 84 high-throughput chemical screen hits were subjected to pharmacophore identification in order to establish the similarities between the hits and characterise the preferred group of lead compounds for further validation and structure-activity relationship (SAR) studies. Each 2D structure was first viewed by eye to distinguish common scaffolds and identify theoretical pharmacophores. Using this approach 13 theoretical pharmacophore groups were identified for 50 of the lead hits, with the remaining 34 hits having no common structures with any other lead hits. The identification of 13 theoretical pharmacophore groups was encouraging, given that no compound was more than 85% similar to any other (Tanimoto dissimilarity *T* value ≤ 0.85).

**Figure 7:**
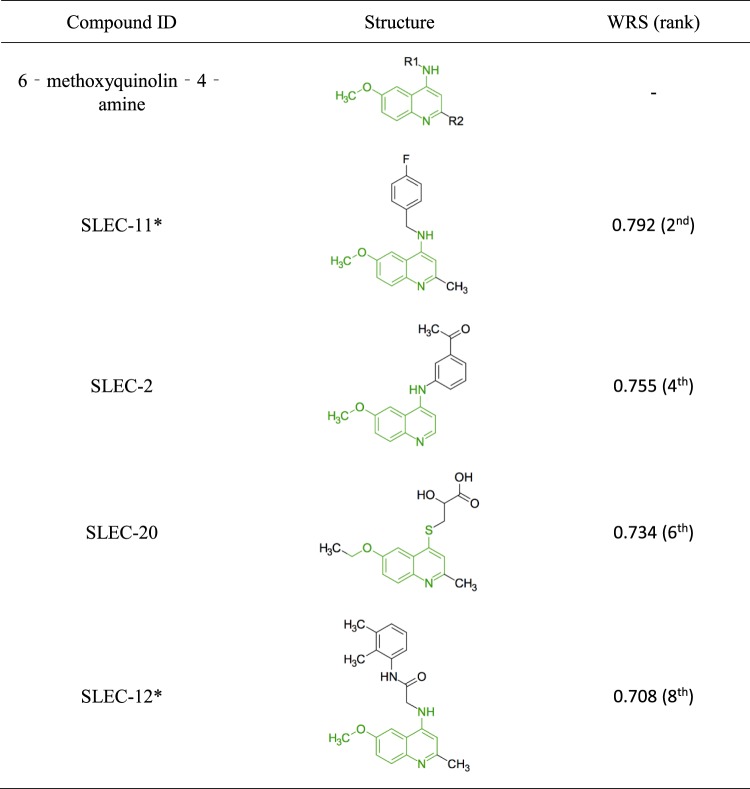
The 6‐methoxyquinolin‐4‐amine theoretical pharmacophore group. The theoretical pharmacophore group is shown in green. The weighted ranked score was calculated as described above from six variables. The rank is shown in parenthesis. Asterisk represents the lead compounds chosen for validation in 96-well plates.

The 6-methoxyquinolin-4-amine pharmacophore group (Fig. 7) was the top pharmacophore group with four compounds in this group ranked in the top eight using the WRS. The 6-methoxyquinolin-4-amine pharmacophore is of a relatively planar geometry because all atoms comprising the quinoline ring system are sp2 hybridized. Therefore, this scaffold can engage in strong Van der Waals interactions and may also fit easily into a narrow hydrophobic binding pocket. Limited SAR could be gleaned from the four compounds shown in Fig. 7 and additional analogues will need to be investigated in future studies. All pharmacophore groups have not been included here due to space constraints, but are available on request.

Generally, only one compound was chosen for validation per pharmacophore, but two leads were selected for the 6- methoxyquinolin-4-amine group (SLEC-**11** and SLEC-**12**) due to the high WRS values. The top pharmacophore groups were selected based on the number and strength of leads within each group. The largest pharmacophore group contained six lead compounds in the top 84 (Supplementary Table 3).

For the validation of lead compounds, nine hits were chosen from the 13 predicted pharmacophore groups and three additional hits without a group were chosen for a total of 12 lead SL compounds covering a significant chemical space (Table 1, Supplementary Fig. 4).

**Table 1:** Medium-throughput validation of the 12 lead compounds.

Compound ID (SLEC-)	Differential at 5 µM (%)	MCF10A WT EC_50_ (μM)	MCF10A *CDH1*^*−/−*^ EC_50_ (μM)	EC_50_ ratio (MCF10A WT/MCF10A *CDH1*^*−/−*^)
1	12.50^†^	38.38	22.64	1.70
6	20.78*	22.56	14.49	1.56
8	22.79*	12.36	9.07	1.36
11	20.74*	10.77	7.02	1.53
12	11.47	13.04	8.98	1.45
13	20.94^†^	10.92	8.77	1.25
14	26.54	15.71	9.84	1.60
15	6.25	29.79	23.18	1.29
16	16.17*	17.05	8.53	2.00
17	3.54	36.40	23.05	1.58
18	18.57*	15.62	10.60	1.47
19	10.75^†^	5.12	4.52	1.13

The differential and EC_50_’s were calculated from at least two biological replicates performed in 96-well plates using Hoechst-33342 and CellProfiler to enumerate counts for end-point analysis as described in the methods. The differential was calculated from the normalized cell counts for MCF10A WT – MCF10A *CDH1*^*−/−*^. EC_50_ concentrations were calculated in Prism using a nonlinear regression curve fit from at least five concentration points. *p-value < 0.05, as determined by a two-tailed equal variance Students T-test. ^†^the differential was calculated at 6.25 µM not 5 µM. SLEC compounds **8** and **11** were chosen for further studies.

### Validating and triaging lead compounds

The top 12 compounds identified from the four-staged HTS campaign (Supplementary Fig. 4) were validated using both real-time and end-point assays in 96-well plates, with the top two compounds prioritised for future SAR studies.

**Figure 8:**
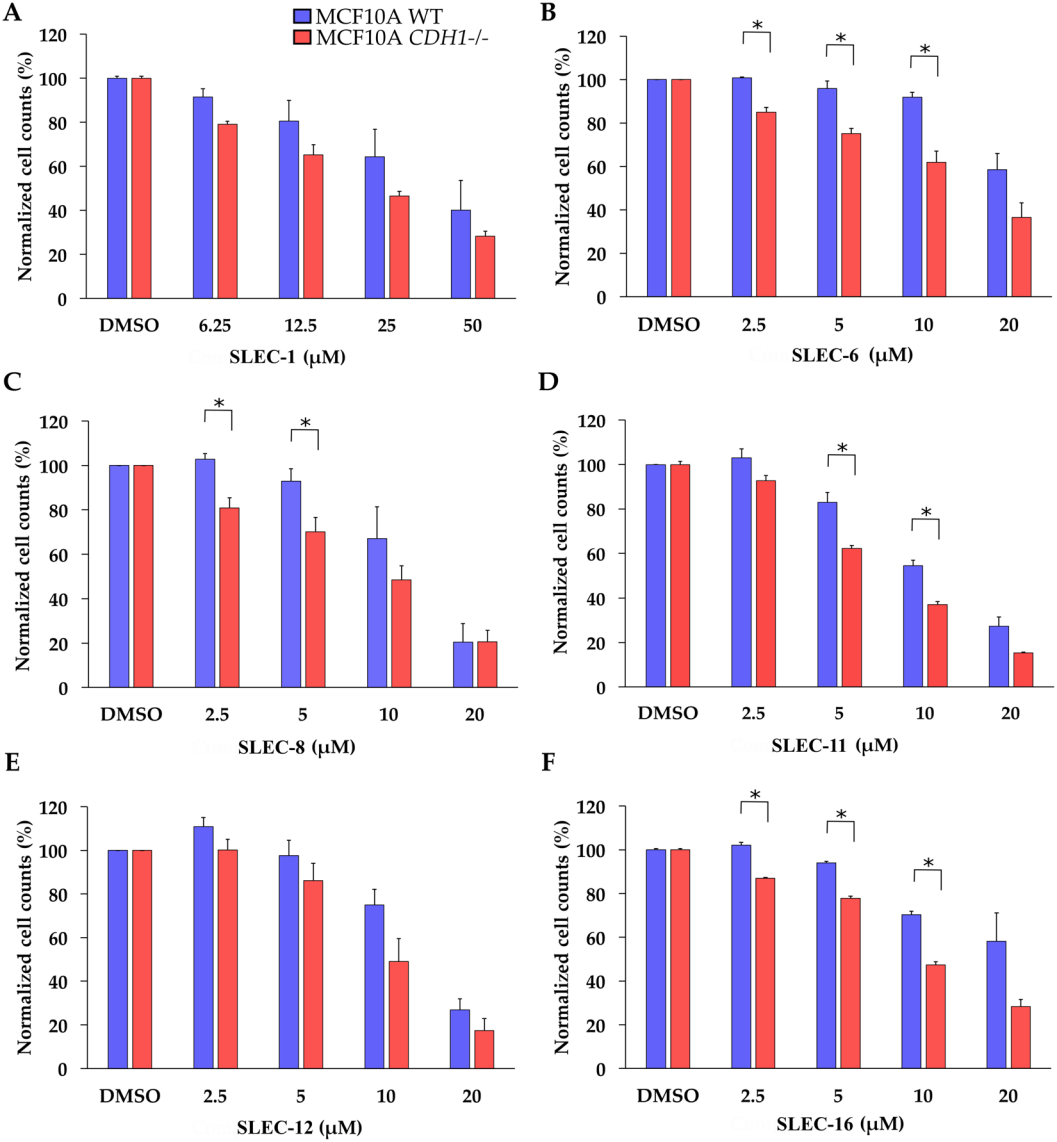
Dose-response validation data for a selection of lead synthetic lethal compounds. MCF10A WT and MCF10A *CDH1*^*−/−*^ cells were seeded 4,000 cells/well in 96-well plates as described in the Methods. Data shown is the mean of at least three biological replicates. Error bars are SEM. *p-value < 0.05, as determined by a two tailed equal variance Students T-test. Only selected concentrations of interest are shown.

There were significant SL differentials for SLEC compounds **6**, **8**, **11** and **16** (Fig. 8). SLEC-8 was chosen as a lead compound for SAR analysis as it had the highest significant SL differentials at 2.5 μM and 5 μM of 22.0% (p = 0.038) and 22.8% (p = 0.014), respectively (Table 1). It also had a relatively low EC_50_ in MCF10A *CDH1*^*−/−*^ cells of 9.1 μM. The second lead compound chosen was SLEC-11; it had the second lowest EC_50_ in MCF10A *CDH1*^*−/−*^ cells of 7.0 μM, the third highest significant differential of 20.7% (p = 0.011) at 5 μM (Table 1) and represented the 6-methoxyquinolin-4-amine pharmacophore group (Fig. 7).

**Figure 9:**
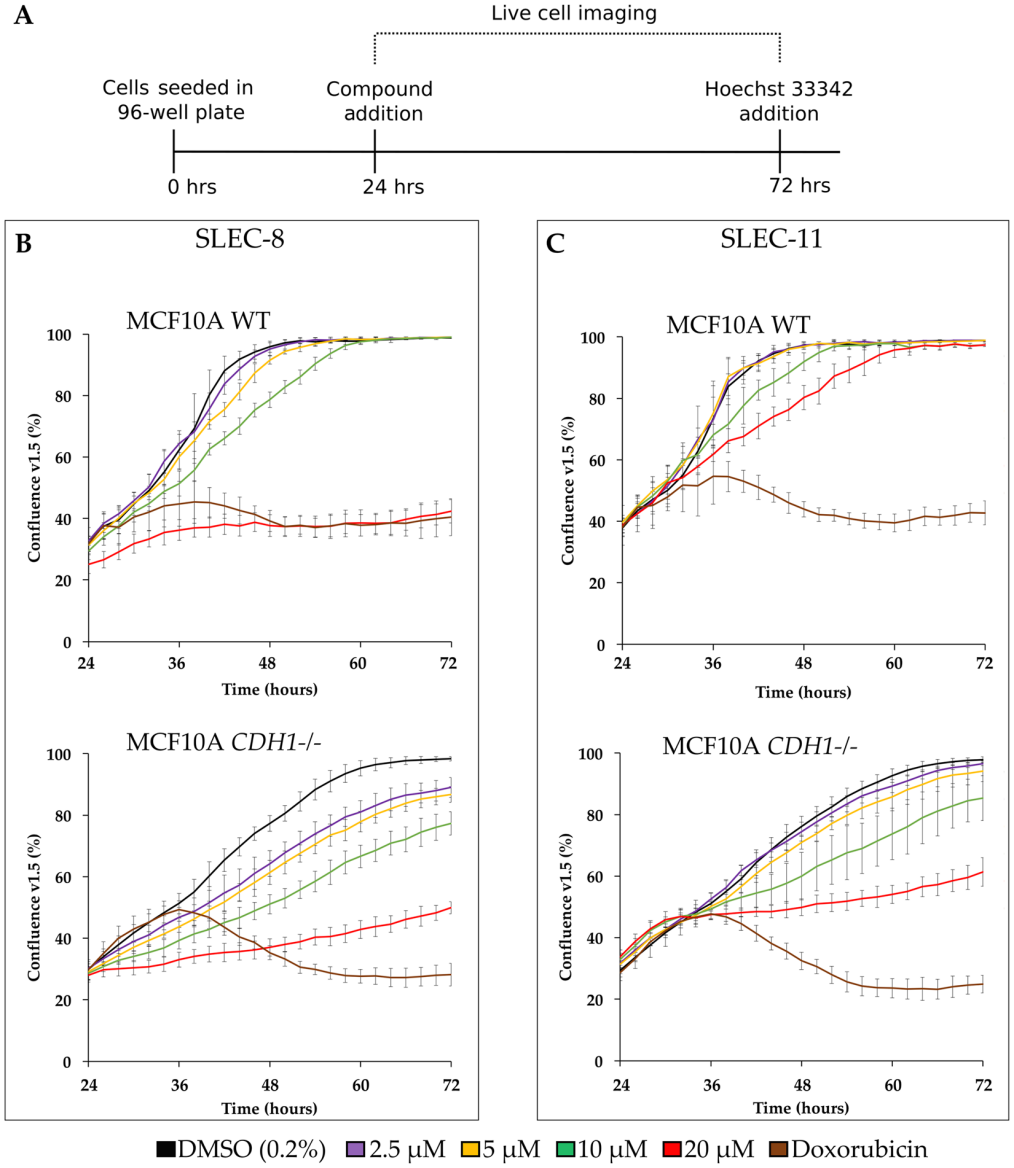
IncuCyte validation data for lead compounds 8 and 11. (**A**) Timeline for the combined live cell imaging and end-point viability assays. Plates were seeded 4,000 cells/well as described in the Methods. (**B,C**) One hour following compound addition, plates were transferred to the IncuCyte. Three 4x images per well were taken every two hours for 48 hours. An EC_80_ dose of doxorubicin was used (Supplementary Table 1). Data shown is a representative experiment of three biological replicates. Error bars are SD. The cell confluence v1.5 was calculated using the IncuCyte software. (**B**) Live cell imaging for SLEC-8. (**C**) Live cell imaging for SLEC-11. Graphs were created in Excel.

Real-time viability assays were performed on the two lead compounds SLEC-8 and SLEC-11 using the two isogenic MCF10A cell lines. The IncuCyte and xCELLigence assay systems quantify cell growth over the drugging period in real-time, allowing for the assessment of even subtle growth inhibitory effects following compound treatment. The IncuCyte was used in combination with an end-point assay (nuclei counting) to give both live cell imaging and normalized cell counts for each plate. For both SLEC compounds **8** and **11**, a dose-dependent growth rate reduction was observed in MCF10A *CDH1*^*−/−*^ cells at all concentrations between 2.5–20 μM (Fig. 9). Critically, a corresponding growth reduction in MCF10A WT cells was only observed at the higher concentrations.

To calculate growth rates, a trendline was fitted to the linear growth phase of each cell line. The greatest SL difference in the slope of the trendline for SLEC-8 as at 10 μM treatment with 2.0 fold difference for MCF10A *CDH1*^*−/−*^ cells, compared to MCF10A WT. For SLEC-11 the greatest SL difference was at 20 μM, with a 4.0 fold difference in the slope of the trendline for MCF10A *CDH1*^*−/−*^ cells compared to MCF10A WT (data not shown).

The xCELLigence real-time system quantifies electrical impedance as a measurement of cell viability which is expressed as the cell proliferation index. A steep drop in the cell proliferation index was observed for all wells within the first 30 minutes of compound addition. This was due to drug effects and briefly removing plates from the incubator for the addition of compounds and controls. Despite a slower adhesion time for MCF10A *CDH1*^*−/−*^, consistent with our previous finding^3^ the DMSO control treated cells had similar slopes of 0.17 and 0.15 for MCF10A WT and MCF10A *CDH1*^*−/−*^, respectively.

**Figure 10:**
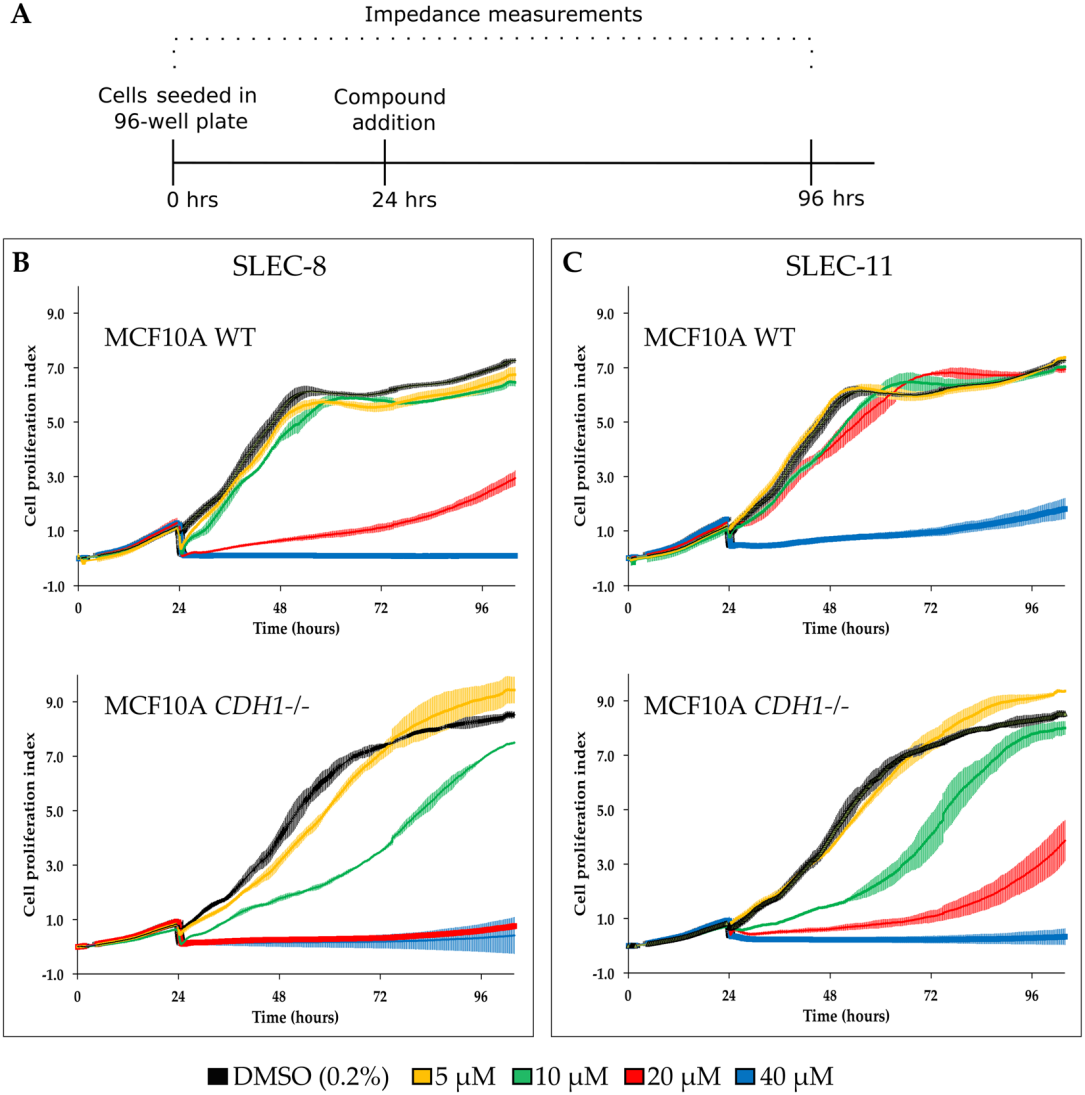
Real-time xCELLigence data for lead compounds 8 and 11. **(A**) Timeline for the real-time cellular assay. Plates were seeded 4,000 cells/well as described in the Methods. (**B,C**) Data shown is a representative experiment of two biological replicates, performed in 96-well xCELLigence E-plates. Error bars are SD. Measurements were taken every 15 minutes. The cell proliferation index was calculated using the RTCA software. (**B**) Cells were treated with SLEC-8. (**C**) Cells were treated with SLEC-11.

Similar to the IncuCyte and Hoechst end-point assays, 10 μM of SLEC-8 caused a significant SL differential between the two cell lines (Fig. 10B). By 60 hours post seeding MCF10A WT cells were able to fully recover, but MCF10A *CDH1*^*−/−*^ were delayed with a 2.7 fold decrease in cell proliferation index for 10 μM compared to DMSO in MCF10A *CDH1*^*−/−*^ cells. Even by 80 hours post drugging MCF10A *CDH1*^*−/−*^ cells had not fully recovered to the same level as DMSO. For SLEC-11 the difference in cell proliferation index between 20 μM and DMSO in MCF10A *CDH1−/−* cells steadily increased to a maximum of 7.9 fold at 62 hours post seeding (Fig. 10C). There was no difference at the same time point in the MCF10A WT cells as they were able to fully recover.

Therefore, similar to the IncuCyte and Hoechst end-point assays, SLEC-11 caused a significant SL differential between the two isogenic MCF10A cells lines. For both compounds the drug effect appeared to wear off around 72 hours post seeding (48 hours post compound addition).

Similar to the cell number end-point assays, after addition of SLEC compounds **8** and **11** the recovery of MCF10A WT, but not MCF10A *CDH1*^*−/−*^ cells to levels similar to that of DMSO was observed in both real-time assay systems. But interestingly, a higher SL differential for both compounds was observed in the real-time analysis. SLEC-8 had an immediate effect on cell viability, whereas SLEC-11 slowly inhibited MCF10A *CDH1*^*−/−*^ cells. The lead compounds therefore appeared to show different dynamics of inhibition. Overall, the combined viability assays demonstrated the increased susceptibility of MCF10A *CDH1*^*−/−*^ cells to both SLEC compounds **8** and **11** compared to MCF10A WT cells.

## Discussion

Current strategies for the chemotherapeutic treatment of E-cadherin-deficient tumors are limited; consequently, there is an urgent need to develop novel compounds for the treatment and/or chemoprevention of these diseases. We hypothesized that *CDH1* loss creates vulnerabilities in a tumor cell that can be specifically targeted with drugs using a synthetic lethal approach. This approach in a therapeutic setting confers a selective advantage to conventional drug approaches as it targets vulnerabilities found only in tumors harbouring specific mutation(s) (e.g. *CDH1*) not found in normal cells.

To discover novel compounds for the treatment of cancer arising from E-cadherin loss, we undertook an unbiased high-throughput phenotypic screen of 113,945 novel lead-like compounds. This resulted in the successful validation of six SL compounds in our model system, two of which have been chosen for future target identification. The novel compounds identified in this four-staged screening campaign had higher EC_50_ fold changes between MCF10A WT and MCF10A *CDH1*^*−/−*^ cells compared to the hits from other E-cadherin SL screens performed by our laboratory^34^ and others^42,43^. This is promising, as it implies that many of the novel lead compounds identified in this HTS could surpass the activity of the currently known drugs we have identified for treating E-cadherin deficient cancers.

One notable finding of this HTS was the substantial number of RSL compounds identified in the single point confirmation screen. The RSL hits also had on average, higher RSL differentials compared to the SL compounds. RSL effects of up to a 20 fold change in EC_50_ have been observed in other E-cadherin SL drug screens^42^. The RSL effects could be explained by the induction of an EMT via E-cadherin loss, making cells more resistant to a subset of drugs^44^. However, we have previously shown that E-cadherin loss alone was insufficient to cause a complete EMT in MCF10A cells^3^. Alternatively, RSL proteins may have functional homologues which are activated in E-cadherin-deficient cells, rendering the RSL protein redundant. As a consequence, *CDH1*^*−/−*^ cells will be less sensitive to inhibition of the RSL protein than wild type cells^39^.

This work aims to provide the foundation for the eventual prevention and treatment of both sporadic and hereditary LBC and DGC. The identification of the novel lead compounds SLEC-8 and SLEC-11 provide an appropriate starting point for designing more potent analogues targeting E-cadherin-deficient cells. To enable efficient SAR-directed compound design and optimisation, target identification will be required. Further testing of the optimised lead compounds in *in vitro* and *in vivo* cancer models will be needed to determine if the observed SL effect will translate to a clinically relevant cancer therapy.

## Methods

### Cell culture

All cell lines were grown in a humidified cell culture incubator at 37 °C and 5% CO_2_ and maintained in specific growth media. MCF10A (CRL-10317) and the derived *CDH1*-negative isogenic line (MCF10A *CDH1*^−/−^; (CLLS1042)) were purchased from Sigma-Aldrich. MCF10A isogenic cells were cultured in a 1:1 mixture of Dulbecco’s modified Eagle’s medium and F12 medium (DMEM-F12) and supplements as described previously^30^.

MCF10A WT and MCF10A *CDH1*^−/−^ cell lines were passaged for no more than 10 passages post freeze-thaw. Automated cell counts for passage calculations were obtained from the CellCountess automated cell counter (Thermo Fisher Scientific). Cells were routinely tested for mycoplasma contamination.

### Novel compound library

The Stage 6 WECC compound library collated by Baell^32^ was stored at −80 °C in a purpose-built small molecule repository. During the high-throughput screen, 384-well library plates (5 mM in DMSO) were stored at −20 °C.

In order to independently validate hits from the HTS lead compounds were purchased from Ambinter (France) and SLEC-11 was synthesized at the Ferrier Research Institute (Wellington, New Zealand) by Dr. Andreas Luxenburger (WO 2017/085053).

The lead compounds SLEC-8 (Supplementary Fig. 5) and SLEC-11 (Supplementary Fig. 6) were characterized by ^1^H and ^13^C nuclear magnetic resonance spectroscopy and electrospray ionization mass spectrometry. Purity of SLEC-11 (Supplementary Fig. 7) was determined by high-performance liquid chromatography (HPLC). Purity of SLEC-8 was determined by HPLC to be greater than 90% (Ambinter, France). All chemical structures were drawn using MarvinSketch. The starting material, 4-chloro-6-methoxy-2-methylquinoline, was prepared following the procedures in Anukumari, *et al*.^45^.

#### N-(4-Fluorobenzyl)-6-methoxy-2-methylquinolin-4-amine (SLEC-11)

To a solution of 4-chloro-6-methoxy-2-methylquinoline (201 mg, 0.968 mmol) in 1,4-dioxane (15 mL) was added (±)-2,2′-*bis*(diphenylphosphino)-1,1′-binaphthyl (rac-BINAP; 181 mg, 0.291 mmol), cesium carbonate (947 mg, 2.91 mmol), 4-fluorobenzylamine (0.16 mL, 1.40 mmol) and *tris*(dibenzylideneacetone)dipalladium(0) [Pd_2_(dba)_3_; 178 mg, 0.194 mmol], and the resulting mixture was heated at 110 °C overnight. The reaction was diluted with water and extracted with ethyl acetate (3×). The combined organic phases were washed with brine, dried over MgSO_4_ and concentrated. The crude product was purified by flash column chromatography (silica gel, ethyl acetate/petroleum ether/triethylamine 3:7:0 then 1:1:1) to yield 134 mg of SLEC-11 (47%) as a colorless, amorphous solid. ^1^H NMR (500 MHz, CDCl_3_) δ 7.86 (d, 9.2 Hz, 1 H), 7.37 (AA′BB′X, J_AB_ = 8.7, J_AF_ 5.4 Hz, 2 H), 7.28 (dd, 9.2, 2.7 Hz, 1 H), 7.07 (AA′BB′X, J_AB_ = 8.7, J_BF_ 8.9 Hz, 2 H), 6.97 (d, 2.7 Hz, 1 H), 6.33 (s, 1 H), 5.14–5.05 (m, 1 H), 4.49 (d, 5.3 Hz, 2 H), 3.87 (s, 3 H), 2.56 (s, 3 H); ^19^F NMR (470 MHz, CDCl_3_) δ −114.63; ^13^C NMR (125 MHz, CDCl_3_) δ 163.31/161.36 (d, 244.7 Hz), 157.11, 156.45, 148.54, 143.89, 133.54/133.51 (d, 2.7 Hz), 130.76, 129.22/129.15 (d, 8.1 Hz) 120.20, 117.75, 115.86/115.69 (d, 21.4 Hz), 100.02, 99.06, 55.63, 46.95, 25.44; HRMS (ESI) m/z calcd for: C_18_H_17_FN_2_OH^+^ 297.1398, found 297.1408.

### High-throughput screen

MCF10A WT and MCF10A *CDH1*^*−/−*^ cells were seeded into Corning black walled, clear bottom 384-well plates (assay plates) using a multidrop 384 reagent dispenser (Thermo Fisher Scientific) and a total volume of 50 μL per well.

As we have shown MCF10A *CDH1*^*−/−*^ cells have a prolonged lag to log phase growth of around 24 hours^3^, in order to achieve a similar confluence at 72 hours post seeding MCF10A WT and MCF10A *CDH1*^*−/−*^ cell lines were seeded at 600 cells/well and 800 cells/well, respectively. Following seeding, plates were left for one hour at RT without stacking^46^ and then centrifuged at 500 × g (RCF) for one minute. Plates were then transferred to an incubator at 37 °C and 5% CO_2_. At 24 hours post seeding, the MiniTrak robotic liquid handling system (Perkin Elmer) was used to transfer 352 compounds per 384-well library plate to the first 22 columns of a 384-well assay plate containing cells to achieve a 10 μM final compound concentration for the pilot, primary and single-point confirmation screens. For the 11-point dose-response screen, compounds were added to columns 1–22, but excluding edge rows A and P of the library plate to reduce edge-effects (Supplementary Fig. 2). The controls of DMSO (0.2% v/v), doxorubicin (EC_80_) and entinostat (EC_50_) were added into columns 23–24 of each assay plate (Supplementary Table 1). Cell titre blue^47^ was made in-house in sterile PBS from 597 μM resazurin, 78 μM methylene blue, 1 mM potassium hexacyanoferrate (III) and 1 mM potassium hexacyanoferrate (II) trihydrate. At 69 hours post seeding, 16.7% v/v CTB was added to plates and they were incubated at 37 °C for 3 hours (Supplementary Fig. 1). At 72 hours post seeding, plates were removed from the incubator and left for 30 minutes at RT to equalise the temperature across all wells of the plates and reduce fluorescence-based edge effects before reading on an EnVision (PerkinElmer) at 550 nm excitation and 590 nm emission to quantify cell viability.

A direct measurement of cell counts using cellular imaging of nuclei was also included at the 11-point screening stage. Plates were fixed, washed and stained using an ELx405 Select deep well washer (BioTek). Media was first aspirated leaving 10 μL/well, then 4% w/v PFA was added and plates left for 15 minutes at RT. Plates were then washed twice with PBS-T, before aspirating all permeabilization buffer and adding Hoechst 33342 (1 μg/mL final) in PBS. Plates were then stored in the dark at 4 °C, until being imaged on a BD Pathway 855. Two 10x images per well were taken and cell counts enumerated using CellProfiler^48^.

### Validation assays

Cells were seeded into each well of Corning’s black walled, clear bottom 96-well plates with a total volume of 100 μL per well. Edge wells were excluded. Both isogenic cell lines were seeded into the same plate to mitigate plate-to-plate variation. Plates for real-time analysis were transferred to the xCELLigence platform (ACEA Biosciences, USA) or the IncuCyte FLR imaging system (Essen BioScience, USA) as previously described^49^.

Seventy-two hours post seeding (or 48 hours post compound addition), plates were assayed for cell viability using a one-step cocktail of PFA (0.25% v/v), saponin (0.075% w/v) and Hoechst 33342 (1 μg/ml final) as described previously^49^. CellProfiler was used to enumerate cell counts as previously described^48^.

### Statistical analysis

With the aim of taking into account the positional effect of samples and to absolve systematic edge effects for the pilot and primary screens which used edge wells, a custom well correction factor was used (Eq. 1). The batch median relative fluorescence unit (RFU) refers to the median of the RFU readouts for all the wells from all plates in a batch, excluding the control wells. The batch median of well reference was the median for each specific well across the batch. For example, the median of well A1 across all plates in a batch (Supplementary Fig. 3), hence allowing for each well in each batch to have a unique correction factor.

1 $$Well\,correction\,factor=\frac{Batch\,median\,RFU}{Batch\,median\,of\,well\,reference}$$

The corrected RFU (coRFU) in Eq. 2 is the edge-effect adjusted RFU readout which was calculated based on the well correction factor from Eq. 1.

2 $$coRFU=Raw\,RFU\times well\,correction\,factor$$

The coRFU values were then used to calculate the normalized readouts of percent of controls (Eq. 3) and the robust Z score (Eqs 4,5), where doxorubicin refers to an EC_80_ dose and DMSO was 0.2% v/v.

3 $$coPOC=100\times \frac{(Sample\,coRFU-\bar{{\rm{x}}}\,doxorubicin\,coRFU)}{(\bar{{\rm{x}}}\,DMSO\,coRFU-\bar{{\rm{x}}}\,doxorubicin\,coRFU)}$$

4 $$MAD=median\,(Sample\,coRFU-plate\,median\,coRFU)$$

5 $$Corrected\,robust\,Z\,score=\frac{(Sample\,coRFU-plate\,median\,coRFU)}{MAD}$$

The B score^36^ was calculated from raw RFU using the R statistical package cellHTS2^50^. The quality control metrics Z factor (Z’)^51^ and strictly standardized mean difference (SSMD)^40^ were used a previously described.

## Supplementary information

Supplementary Information

## Data Availability

The datasets generated during and/or analyzed during the current study are available from the corresponding author on reasonable request.
