# Supplementary material for: A high-throughput screen to identify novel synthetic lethal compounds for the treatment of E-cadherin-deficient cells
Source: Sci Rep. 2019 Aug 29;9:12511. doi: 10.1038/s41598-019-48929-0 (PMC6715681; doi:10.1038/s41598-019-48929-0)
Supplement: Supplementary file 1 — Supplementary Information [file 41598_2019_48929_MOESM1_ESM.pdf]

SUPPORTING INFORMATION

A high-throughput screen to identify novel synthetic lethal compounds for the treatment of E-cadherin-deficient cells

AUTHORS

Henry Beetham[1], Augustine Chen[1], Bryony J. Telford[1], Andrew Single[1], Kate E. Jarman[2-3], Kurt Lackovic[2-3], Andreas Luxenburger[4], Parry Guilford[1,*]

[1] Cancer Genetics Laboratory, Department of Biochemistry, University of Otago, Dunedin, New Zealand

[2] Division of Systems Biology and Personalized Medicine, Walter and Eliza Hall Institute of Medical Research, Parkville, Victoria, Australia

[3] Department of Medical Biology, University of Melbourne, Victoria, Australia

[4] Ferrier Research Institute, Victoria University of Wellington, Lower Hutt, New Zealand

[*] Corresponding Author: Parry Guilford, Cancer Genetics Laboratory, Department of Biochemistry, University of Otago, P.O. Box 56, 710 Cumberland Street, Dunedin, 9054, New Zealand. Email: parry.guilford@otago.ac.nz

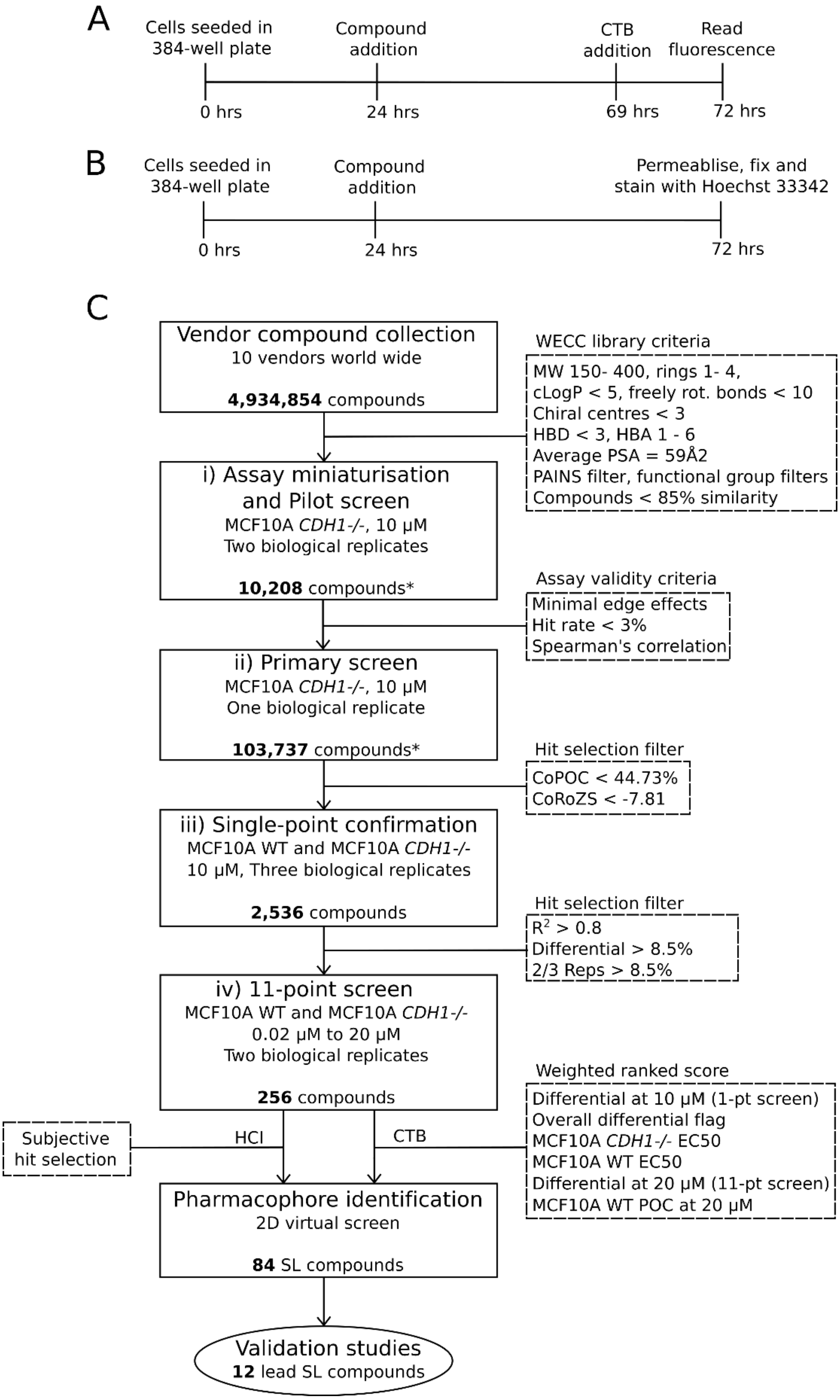

Supplementary Figure 1. High-throughput chemical screen design. A) Timeline for the CTB viability assay. B) Timeline for the HCI assay. C) Overview of the various stages of the screen.*, these compounds are from the WECC library screen described in(33).

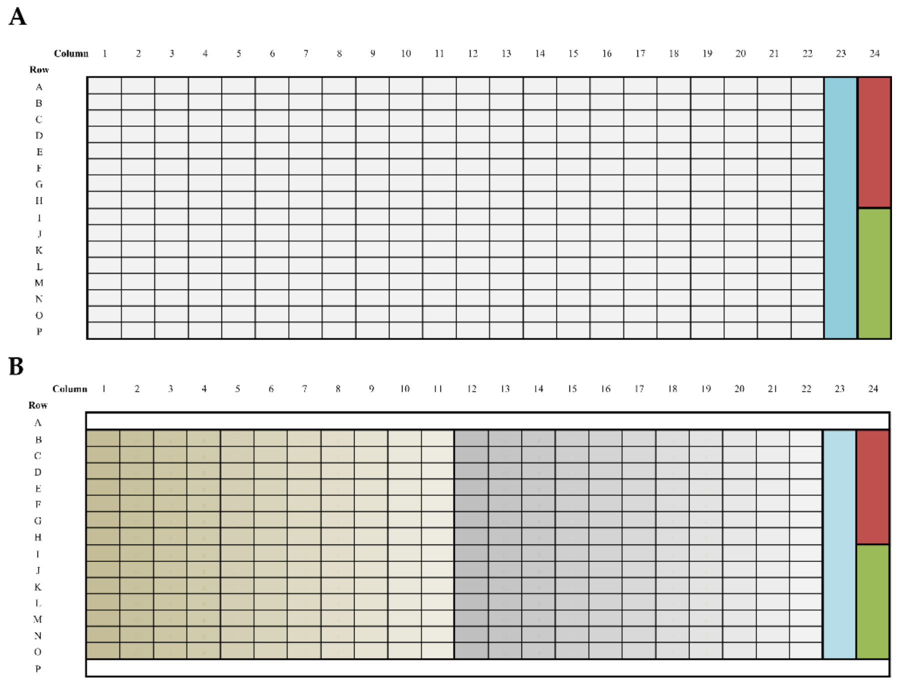

Supplementary Figure 2. **Plate layout for the HTS.** Blue wells represents DMSO (0.2%); red wells, doxorubicin (EC_{80}); green, entinostat (EC_{50}); and grey or gold, WECC compounds. A) Plate layout for pilot, primary and single-point confirmation screens. B) Plate layout for the 11-point screen.

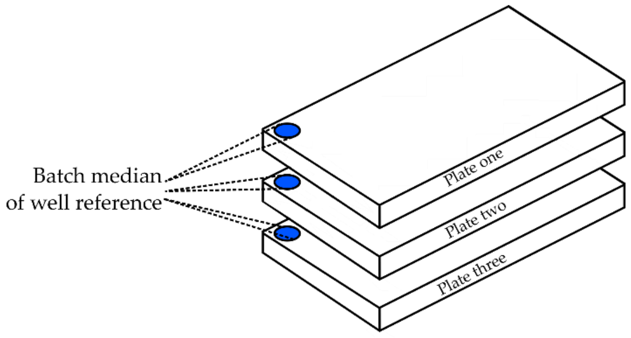

Supplementary Figure 3. Batch median of well reference for well A1.

Supplementary Table 1. Killing control and SL control effective concentrations.

	MCF10A WT	MCF10A *CDH1*$^{-/-}$
Doxorubicin EC_{80}	0.3 μM	0.4 μM
Entinostat EC_{50}	3.2 μM	2.4 μM

MCF10A WT cells were seeded at 600/well and MCF10A *CDH1*$^{-/-}$ cells at 800/well in 384-well plates. Drugs were added at 24 hours post seeding and CTB was used as an end-point assay. Concentrations were normalized to 0.2% v/v DMSO. EC_{50} and EC_{80} curve fits were calculated from 11-point dose response curves at 72 hrs post seeding using Prism 5.

Supplementary Table 2. Raw data before scoring for the top ten compounds from the WRS.

ID (SLEC-)	Differential at 1-pt	Overall Differential flag	MCF10A *CDH1* $^{-/-}$ EC_{50}	MCF10A WT EC_{50}	Differential at 20 μM	MCF10A WT POC at 20 μM	**Sum of Weighted Variables**	Final Rank
**1**	48.04	3.00	12.45	20.00	68.53	98.09	**0.80**	1
**11**	17.42	3.00	6.96	20.00	106.45	100.37	**0.79**	2
**19**	24.41	3.00	7.63	20.00	74.07	93.62	**0.77**	3
**2**	39.99	3.00	3.21	17.66	49.66	42.29	**0.76**	4
**13**	28.81	2.00	10.18	20.00	87.70	98.07	**0.74**	5
**20**	23.81	3.00	9.38	20.00	70.17	92.94	**0.73**	6
**21**	26.17	3.00	12.36	20.00	80.88	99.04	**0.72**	7
**12**	13.92	2.00	3.06	19.41	65.85	44.97	**0.71**	8
**14**	30.75	2.00	11.89	20.00	69.49	88.84	**0.70**	9
**18**	28.37	3.00	9.03	20.00	56.15	62.00	**0.70**	10

Compounds SLEC-1 (blue) and SLEC-11 (red) are also shown in Figure 6. Other compounds of interest are highlighted in green. All data was averaged between the three or two biological replicates, for single-point and 11-point screens respectively. For EC_{50} values > 20, a value of 20 was used for simplicity.

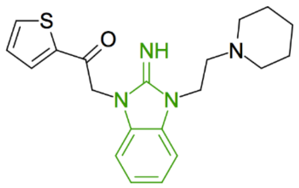

SLEC-1

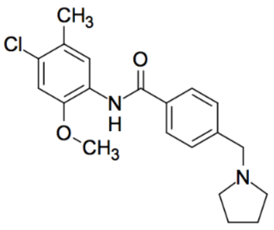

SLEC-6

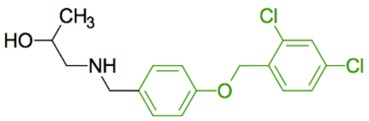

SLEC-8*

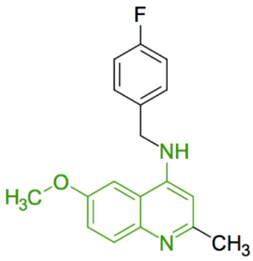

SLEC-11*

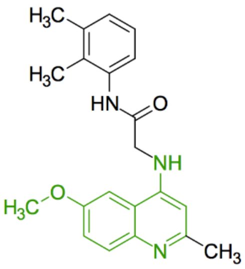

SLEC-12

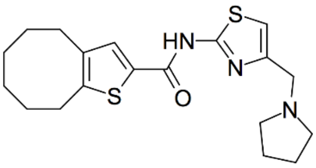

SLEC-13

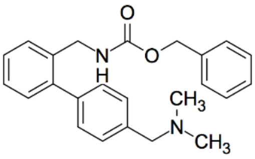

SLEC-14

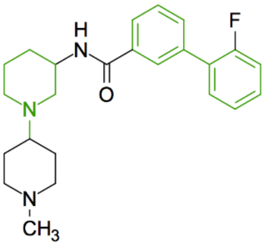

SLEC-15

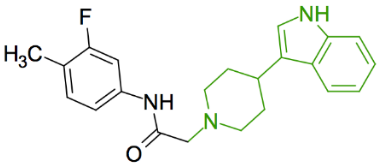

SLEC-16

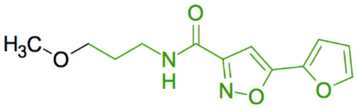

SLEC-17

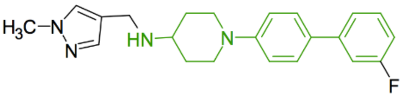

SLEC-18

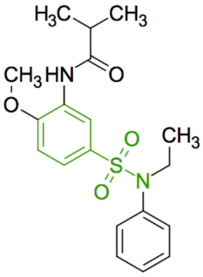

SLEC-19

Supplementary Figure 4. Structures of the 12 lead compounds chosen for validation. The theoretical pharmacophore groups are shown in green. The asterisk highlights SLEC compounds **8** and **11** which were chosen for SAR studies.

Supplementary Table 3. Predicted pharmacophore group for the 1-(4-phenylphenyl)piperidin-4-amine group.

Compound ID (SLEC-)	Structure	WRS (rank)
**18***	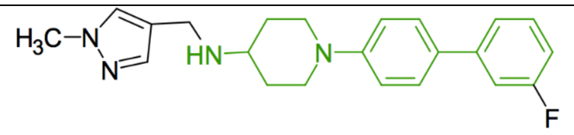	0.696 (10th)
**24**	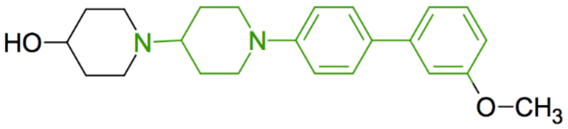	0.677 (19th)
**25**	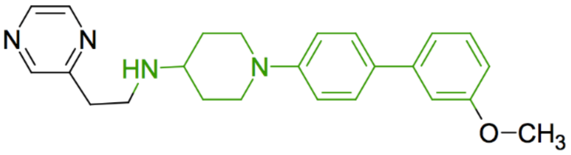	0.676 (20th)
**26**	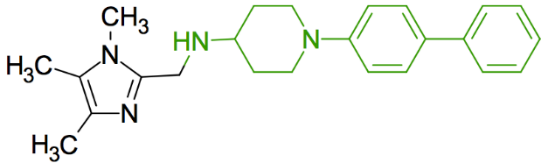	0.661 (25th)
**27**	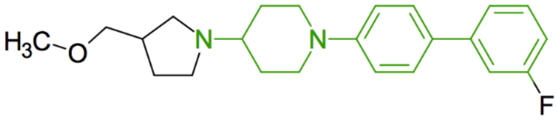	0.596 (64th)
**28**	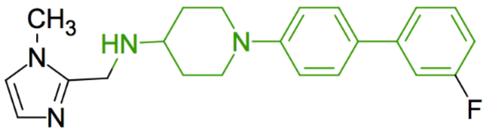	0.567 (83rd)
**29**	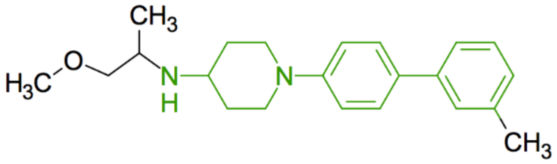	0.474 (193rd)
**30**	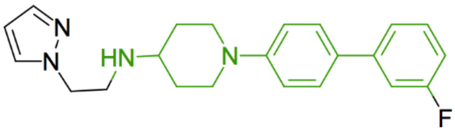	(-4.3%)

The theoretical pharmacophore group is shown in green. WRS, calculated as described above from six variables. Rank is shown in parenthesis, but if compounds were not in the top 256, data for POC differential from the single-point confirmation screen is shown. Asterisk represents the lead compounds chosen for validation in 96-well plates.

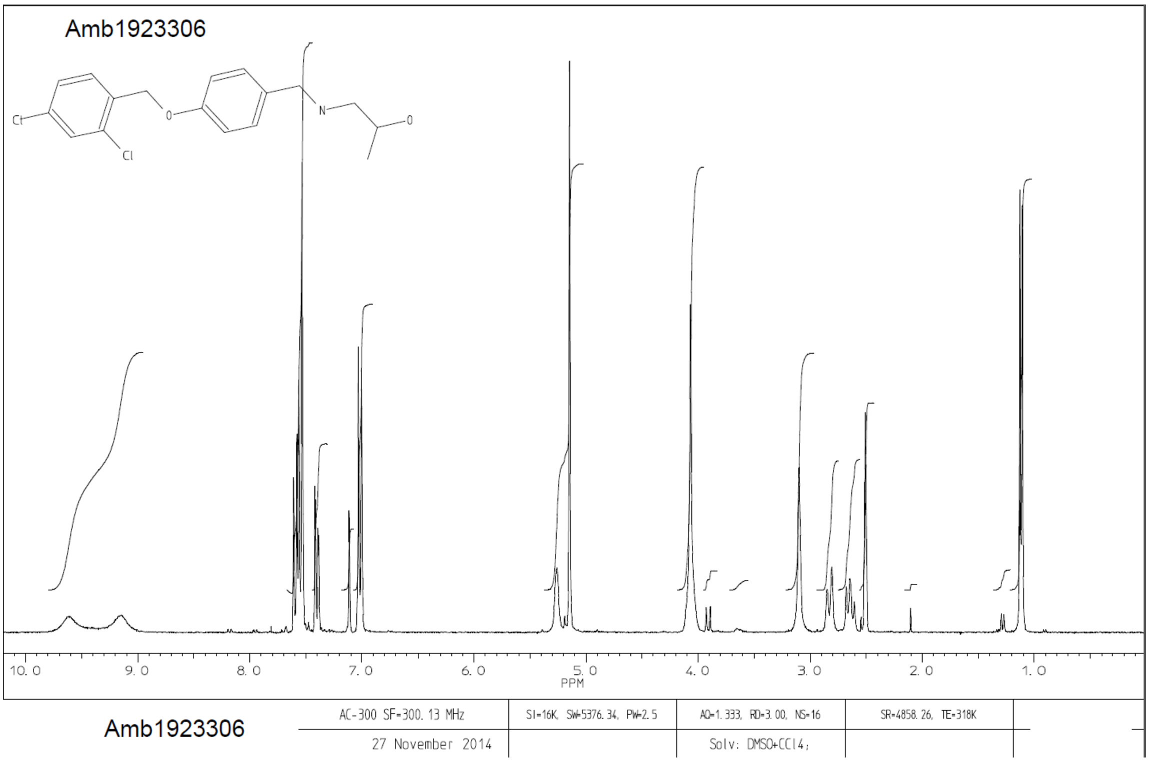

Supplementary Figure 5. **SLEC-8 NMR analysis.** ^{1}H and ^{13}C nuclear magnetic resonance spectroscopy for SLEC-8 (Amb1923306) was determined by Ambinter (France).

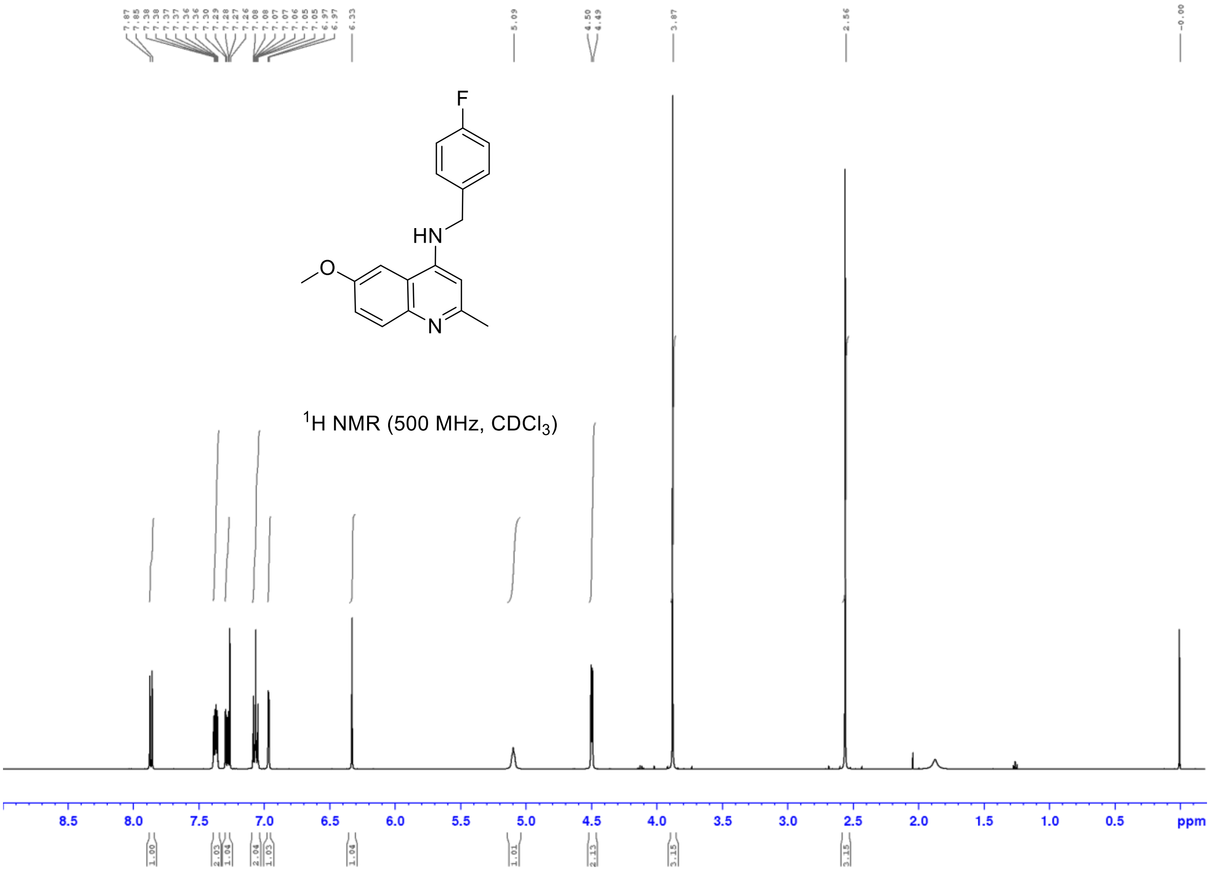
HN
O
N
F
^{1}H NMR (500 MHz, CDCl$_3$)
ppm

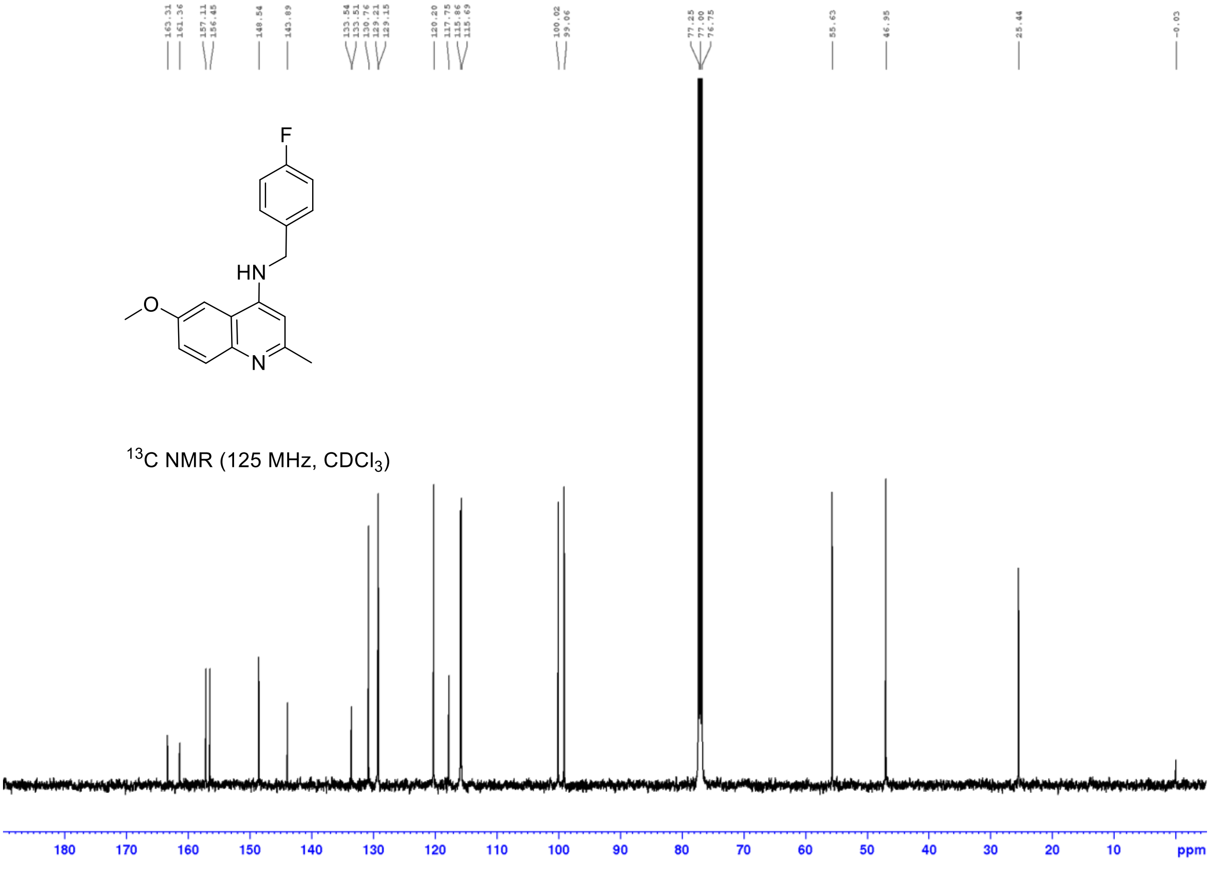
HN
O
N
F
^{13}C NMR (125 MHz, CDCl$_3$)
ppm

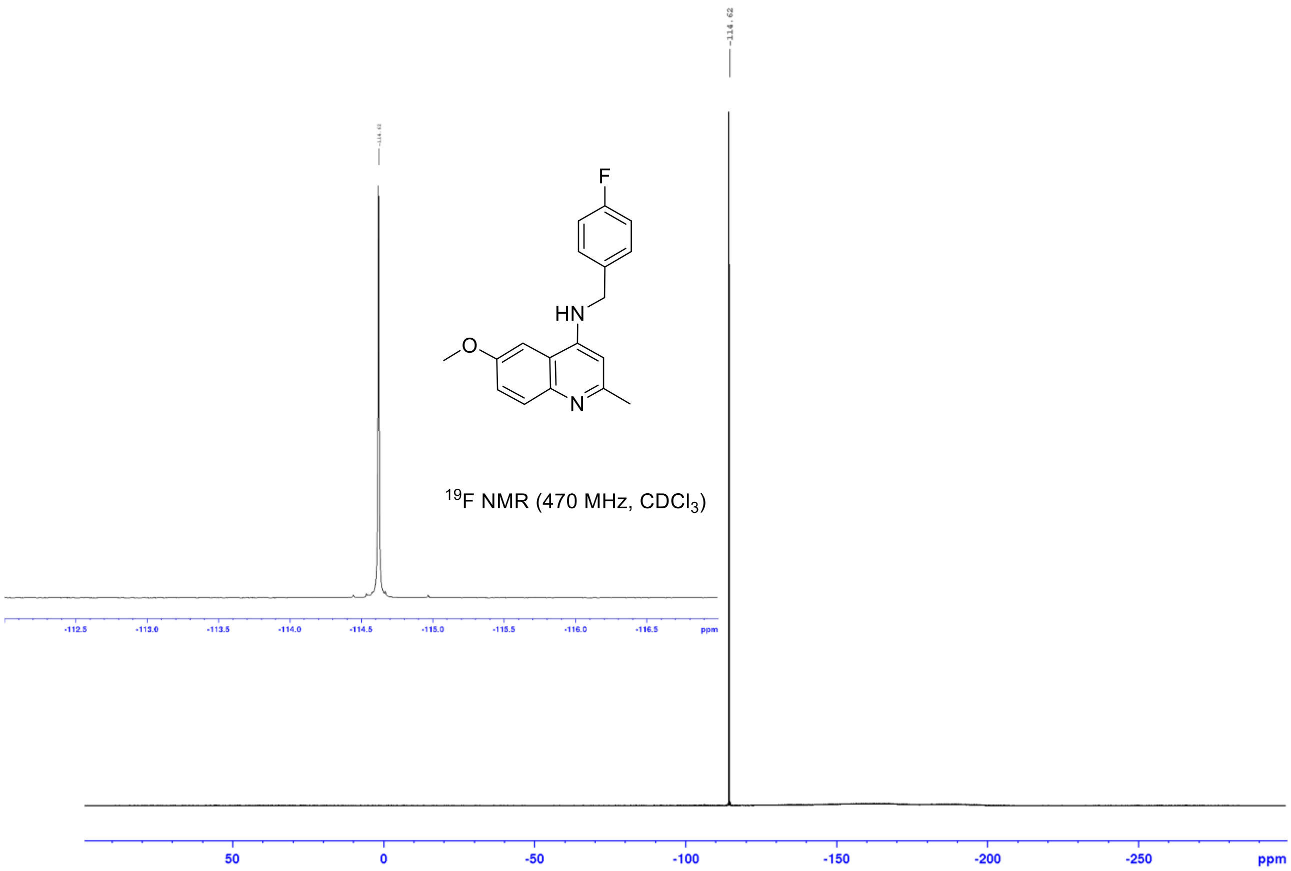

Supplementary Figure 6. ***N*-(4-Fluorobenzyl)-6-methoxy-2-methylquinolin-4-amine (SLEC-11)**. ^{1}H, ^{19}F and ^{13}C NMR spectra. NMR-spectra were recorded on a Bruker Avance (III)-500 spectrometer. The ^{19}F NMR spectrum was recorded at 470 MHz and is reported unreferenced. Chemical shifts are reported in ppm relative to Me_4Si (TMS, δ 0), or the residual solvent peak as an internal standard set to δ 7.26 and 77.00 ($CDCl_3$).

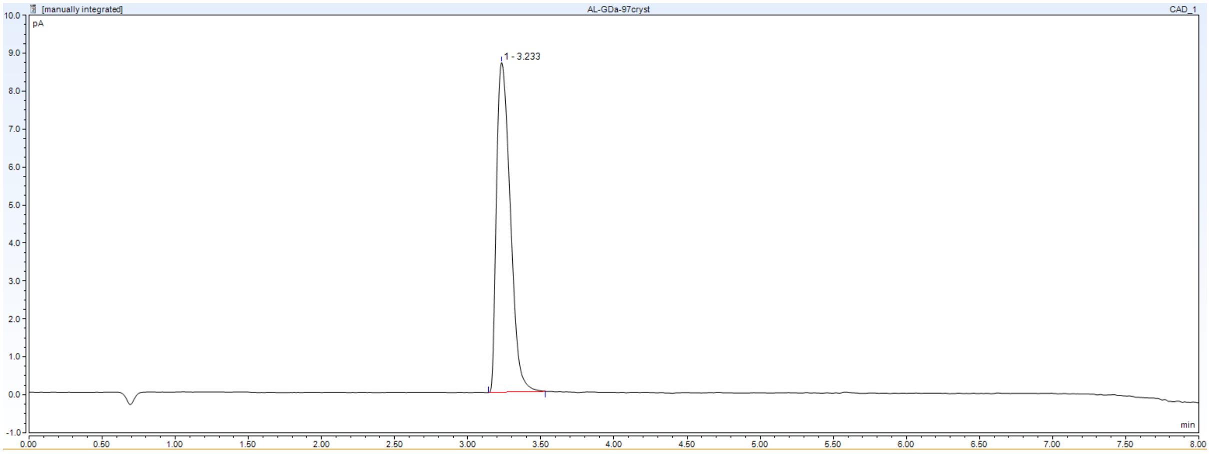

Integration Results							
No.	Peak Name	Retention Time min	Area pA*min	Height pA	Relative Area %	Relative Height %	Amount n.a.
1		3.233	0.951	8.690	100.00	100.00	n.a.
Total:			0.951	8.690	100.00	100.00	

Method conditions:

Column: Kinetex C18 2.6 µm 100 Å 100x3 mm
Guard: C18 Security guard ultra
Mobile Phase A: Water/Trifluoroacetic acid (0.05%)
Mobile Phase B: Methanol/Trifluoroacetic acid (0.05%)
Gradient: T0=60:40, T1:50=40:60, T5:50=0:100, T7:50=0:100, T8=60:40
Flow Rate: 0.7 mL/min
Sample Solvent: Methanol
Detection: CAD
Column Temp: 40 °C
Injection volume: 1 mL

Supplementary Figure 7. **HPLC trace of SLEC-11.** High-performance liquid chromatography (HPLC) analysis was performed on an Agilent 1100 (Quaternary pump) HPLC system (Wellington, New Zealand) with a diode array detector (200–400 nm) and a Corona charged aerosol detector (CAD), employing columns as indicated above. Data was processed with Chromeleon™ chromatography data system software 7.2.
